# High prevalence of arthralgia among infants with Chikungunya disease during the 2019 outbreak in northern region of the state of Rio de Janeiro

**DOI:** 10.3389/fped.2022.944818

**Published:** 2022-10-19

**Authors:** Patrícia Damião Gomes, Rayane Figueiredo Silva Moreira Carvalho, Milena Moulin Massini, Rafael Hauaji Garzon, Pollianny Louzada Schiavo, Regina Célia de Souza Campos Fernandes, Thaís Louvain de Souza

**Affiliations:** ^1^ Faculdade de Medicina de Campos, Campos dos Goytacazes, Campos dos Goytacazes, Rio de Janeiro, Brazil; ^2^ Hospital Plantadores de Cana, Campos dos Goytacazes, Campos dos Goytacazes, Rio de Janeiro, Brazil; ^3^ Molecular Identification and Diagnosis Unit, Laboratory of Biotechnology, Center for Biosciences and Biotechnology, Universidade Estadual do Norte Fluminense Darcy Ribeiro, Campos dos Goytacazes, Rio de Janeiro, Brazil

**Keywords:** arbovirus, Chikungunya, children, infants, arthralgia

## Abstract

**Introduction:**

In a low-income setting with simultaneous presence of Dengue virus, Zika virus, and Chikungunya virus (CHIKV) in the same region, the difficulty of establishing a clinical diagnosis when the molecular test is not a possibility. Thus, it is important to identify signs and symptoms of Chikungunya that can be used to differentiate it from other arboviruses in children.

**Methods:**

This is a cross-sectional study, which was developed in Rio de Janeiro State, Brazil, with the analysis of pediatric medical records regarding arboviruses. Considering that the population had already been exposed to Dengue and Zika viruses and were experiencing the first notification of the CHIKV. The ethics committee approved this research, and all those legally responsible for the children signed the consent form.

**Results:**

In total, 159 children were seen of which 98 were suspected CHIKV cases, and 51 had their diagnosis confirmed with reagent IgM/IgG for CHIKV. The symptoms that the pediatric population with CHIKV presented most often were fever (90.2%), arthralgia (76.5%), and exanthema (62.7%) in both suspected and confirmed cases of Chikungunya. Thus, CHIKV in those children presents a clinical profile similar to those found in other studies referring to adults. Additionally, only arthralgia and a high aspartate transaminase were related to the positivity of serology for Chikungunya.

**Conclusions:**

This study describes the signs and symptoms of CHIKV exhibited in the pediatric population with a mild and moderate presentation similar to the findings in the adult during an epidemic experienced in a population vulnerable to CHIKV.

## Introduction

Chikungunya fever is an emerging disease caused by the Chikungunya arbovirus (CHIKV), which belongs to the genus Alphavirus and Togaviridae. CHIKV is transmitted to humans through the bite of the vectors *Aedes aegypti* and *Aedes albopictus* (1). In Brazil, 177,845 cases of CHIKV were reported in 2019, with the southeast region having the highest number of cases, 113,228 (63.7%). Compared with the country's total and within the Southeast region, the state of Rio de Janeiro was the most affected by the Chikungunya outbreak (2). This outbreak was reflected in the city of Campos dos Goytacazes, the third-largest city in the state of Rio de Janeiro, which notified 8,460 cases, resulting in an incidence of 1,667 cases per 100,000 inhabitants in the city (2).

Although the first case of CHIKV in Brazil was reported in the state of Rio de Janeiro in 2010 (3), it was only in 2014 with the detection of several imported and autochthonous cases in the north and northeast of the country that an emergency scenario was identified (4). In 2018, a serological study conducted in Rio de Janeiro showed that 18% of the population has IgM and/or IgG anti-CHIKV, and as arboviruses are prevalent in the country, double-seropositivity for CHIKV and Dengue virus or CHIKV and Zika virus is not uncommon (5).

The clinical manifestations are well-described in adults as persistent fever for an average of 5 days, rash, arthralgia, myalgia, joint edema predominantly in the lower limbs, headache, emesis, and abdominal pain (1). Unlike the adult population, pediatric patients are less likely to develop myalgia and arthralgia (6) and may exhibit a greater variety of dermatological manifestations, such as skin lesions ranging from maculopapular to vesicular–bullous exanthema (1). The most severe neurological (7), cardiac, renal, and hepatic complications including death in children are rare and practically restricted to infants and children with some pre-existing comorbidity (6).

The presence of IgM by ELISA and/or CHIKV RNA RT-PCR are tests performed to confirm the diagnosis of CHIKV against other differential diagnoses in febrile children. Anti-CHIKV IgM can be detected as early as day 2 of the disease; however, even in the acute phase, anti-CHIKV IgG can also be detected in at least 50% of children (8). In scenarios like in Brazil with the simultaneous presence of Dengue virus, Zika virus, and CHIKV and the difficulty in correctly identifying which arboviruses are responsible for the clinical picture of the child, it is important to verify the presence of IgM/IgG for the three types starting on the 6th day.

Considering the epidemiological situation of 2019, the pediatric population in Campos dos Goytacazes city, Rio de Janeiro state, Brazil, was vulnerable to Dengue virus, Zika virus, and CHIKV, and the early diagnosis regarding clinical aspects arising from these viruses was not possible with the absence of molecular test. Therefore, the pediatricians considered the clinical and laboratory findings in formulating a diagnostic hypothesis for Zika, Dengue, and Chikungunya in the only ambulatory specialized in arboviruses in the city. Based on the serological confirmation of CHIKV by IgM and/or IgG from day 6 onward, this cross-sectional study was conducted to characterize the prevalence of clinical and laboratory findings in pediatric patients with mild to moderate Chikungunya.

## Materials and methods

### Design

This was observational, cross-sectional research conducted in the municipality of Campos dos Goytacazes located in the northern portion of the state of Rio de Janeiro, with an analysis of pediatric medical records generated in the only public health unit specializing in arboviruses in the city in the first half of 2019, the year of the highest incidence of arboviruses in the interior of the state of Rio de Janeiro (2).

All patients were seen by the same PLS pediatrician, and the data from medical records were collected by PDG, RFSMC, MMM, and RHG. The form used for the collection was composed of closed questions with the extraction of secondary data from medical records such as age, gender, signs, and symptoms (a), laboratory test results (b), diagnostic hypotheses (CHIKV, Dengue), and final diagnosis (CHIKV, Dengue).

Secondary data collection (a): fever, rash, conjunctival hyperemia, arthralgia, arthritis, myalgia, headache, retroorbital pain, hemorrhage, edema, sore throat, vomiting, neurological manifestation, irritability, drowsiness, abdominal pain, purpuric lesions, vesicular lesions, bullous lesions, lymph node enlargement, pruritus, cough, oral sores, bitter taste in the mouth, anorexia, asthenia, diarrhea, nausea, and lower limb pain.

Laboratory test results (b): leukopenia, hemoconcentration, aspartate transaminase (AST) elevated, erythrocyte sedimentation rate (ESR) elevated, thrombocytopenia, c-reactive protein elevated, microcytosis, anemia, and diagnosis of Dengue and Chikungunya were made using NS-1 antigen and IgM/IgG ELISA for Dengue and Chikungunya.

Inclusion criteria: age between 1 day and 14 years. The exclusion criterion was children who received diagnostic hypotheses other than arboviruses. Statistical analyses were performed using R software (Vienna, Austria), and 5% significance was considered in the statistical tests. The exposure variables used were age, inflammatory markers, changes in blood count, signs, and symptoms. The outcome variable was the confirmation of infection by the CHIKV in children through testing with reagent results for IgG and/or IgM.

### Ethical aspects

This study was analyzed and approved by the ethics and research committee of the Faculdade de Medicina de Campos (CAAE 72995717.9.0000.5244) and approved by the Clinical Manager of the Reference Center for Immuno-infectious Diseases, meeting the guidelines and criteria established in Resolution 466/12 of the National Health Council. Therefore, all legal guardians of the children signed the consent form.

### Data analysis

Before the processing and analysis of the data obtained, a pre-processing of the variables used was performed, thus the blank fields or fields with an ignored code for age, signs and symptoms, sex, ESR/CRP, and blood count alterations were excluded. Data from children with serological confirmation of Dengue were also excluded.

After the database was created in the Epidata 3.1 software, the data were quantitatively analyzed using the free *software* R (9). Age was the only variable presented as the mean ± standard deviation. The others were presented as the median with the first and third interquartiles. For the descriptive variables, the chi-square test was used, and the chance ratio with a CI of 95% was noted. For continuous variables with distributions outside the normal distribution, the nonparametric Wilcoxon test was used to compare the samples.

## Results

During data collection, 159 children with febrile conditions were seen, 85 of whom were female (53.5%). Of these, 126 had a diagnostic hypothesis of CHIKV and/or other arboviruses of mild to moderate presentation not requiring hospitalization. Of every 10 children with a CHIKV diagnosis, nine had a fever. More than half of the children with the hypothesis of CHIKV presented with fever, exanthema, and arthralgia. Only one child presented neurological manifestation, but was seronegative for Dengue virus and CHIKV.

Outpatient follow-up was determined for all 126 cases, but only 98 returned for serology after 6 days of symptom onset. All 98 children had serological tests for Dengue virus and CHIKV, the mean age was 7.259 ± 3.788 years, and 54 (55.1%) were female. Only three children (3.06%) had reagent serology for Dengue virus, one with reagent IgG and two with IgM. The prevalence of IgM+ anti-CHIKV antibodies was 35%, while IgG+ anti-CHIKV antibodies was 17%. There is no statistical difference between sex and reactive serology for CHIKV (*p *= 0.5149), but patients with reactive serology for CHIKV+ have a higher mean age of 8.408 ± 3.096 years than the patients with the nonreactive serology of 6.011 ± 4.097 (*p *= 0.0043).

The most prevalent signs and symptoms in the 51 children with serologically confirmed CHIKV were the same compared to the symptoms of the 126 children who received the diagnostic hypothesis of CHIKV. Fever, exanthema, and arthralgia were the most common signs, followed by pruritus, headache, and emesis. Of the patients with confirmed CHIKV infection, only arthralgia (OR: 3.391, 95% CI: 1.430–8.044, 0.0089) and elevated AST (OR: 2.688, 95% CI: 1.124–6.430, *p* = 0.0412) were significantly associated with anti-CHIKV seropositivity (IgM+ or IgG+) (Table 1).

**Table 1:** Prevalence of signs and symptoms of the 98 children with a diagnostic hypothesis of CHIKV.

	Suspected cases (*N* = 98)		Confirmed cases (*N* = 51)		
	*N*	(%)	*N*	(%)	*p*
Gender					0.5150
Female	54	55	26	51	
Male	44	45	25	49	
CHIKV	51	52			0.0043
IgM+	34	35	34	66.70	
IgG+	17	17	17	33.30	
Fever	88	90	46	90.20	0.8916
Arthralgia	62	63	39	76.50	0.0089
Rash	57	58	32	62.70	0.4515
Myalgia	15	15	10	19.60	0.3415
Headache	38	39	20	39.20	1.000
Vomit	19	19	19	37.30	0.5703
Retroorbital pain	5	5	1	2	0.3112
Leukopenia	7	7	6	11.80	0.1448
Elevated AST	34	35	23	45.10	0.0412
Elevated ESR	39	40	24	47	0.1856
Itching	42	43	20	39.20	0.5792
Limb pain	20	20	13	25.50	0.2940
C-reactive protein (median)	2.660			3.065	0.5608

CHIKV, Chikungunya; AST, aspartate transaminase; ESR, erythrocyte sedimentation rate.

## Discussion

Typically, the clinical presentation of CHIKV infection in children may be mild to moderate. The risk of evolution to more severe clinical pictures, including neurological involvement and even death, is higher in children under 1 year of age and/or those with previous comorbidities (10). However, in a typical outpatient setting, pediatricians will be faced with mild and moderate cases requiring attention regarding the differential diagnosis with febrile illnesses, especially in regions with other arboviruses, such as Dengue virus and Zika virus. Considering that the population of this study was not previously exposed to CHIKV, despite decades of exposure to Dengue virus (serotypes 1, 2, 3, and 4) and 3 years of notification of Zika virus, in the context of CHIKV outbreak in Campos dos Goytacazes, the first diagnosis hypothesis is mainly CHIKV. These hypotheses are always tested with the serology in ambulatory units, unfortunately, the molecular test for CHIKV are restricted to research settings.

In adults, arthralgia is as prevalent as high fever. In children it is described that arthralgia may be present in 30%–50% of child cases (6), being less frequent in cases infected by the Asian viral strain (11). In this study, all children were evaluated by the same pediatrician, and a higher prevalence of arthralgia than that in the literature was identified; 39 out of 51 children (76.5%) had the clinical picture. In a study carried out with 14 Brazilian children with a mean age of 4 months, the prevalence of joint involvement was 42.8% (1). Since the mean age of the cases in this study is higher, the presentation may be more similar to that of adults, in which fever, arthralgia, myalgia, and headache are more prevalent when compared to children under 1 year of age. Another possible explanation for this study's high prevalence of arthralgia is that the older children can easily complain about this symptom but the younger. However, the pediatrician involved in this study performed a detailed physical examination to identify and record swollen and tender joints in all children.

Elevated levels of liver enzymes are widely described in adult patients (12, 13), and they are among the laboratory abnormalities described in children in addition to leukopenia, thrombocytopenia, and hypocalcemia (6). In a study of 50 children from the French Indian Ocean island Mayotte, elevated AST was found in 24% of cases (14). In the present study, almost twice as many children with high AST were found, 23 out of 51 cases (45.1%) confirmed with CHIKV have this elevation of liver enzymes. The increased prevalence of AST identified here suggests hepatic involvement of the virus in this age group, which has already been recommended in other studies and seems to be even more prevalent in children with severe cases (1). Additionally, self-medication, which is not uncommon in the Brazilian population with the indiscriminate use of nonsteroidal anti-inflammatory drugs to reduce fever, would not alone explain this increase in liver enzyme concentrations.

This study has some limitations, one of them was based on a secondary analysis of medical records. Therefore, some data losses were related to the absence of notes in the medical records, and cases were excluded from the final analysis, reducing the sample size. The objective was exclusively for the outpatient unit specialized in arboviruses. The primary presentation would be mild and moderate clinical pictures because pediatric patients with Chikungunya with acute neurological impairment tend to seek the pediatric emergency room located in another public hospital.

However, it is the only reference outpatient clinic in the city that has more than 500,000 inhabitants, and 2019 was the year of the highest notification of CHIKV cases in the city. The first year of notification in the national grievances system was 2017 with 41 cases, followed by 7657, 8460, 909, and 167 cases in 2018, 2019, 2020, and 2021, respectively. Additionally, in the first year of the CHIKV outbreak in the city, the IgG serologies of these children cannot be explained by previous infections. Therefore, the presented results highlight that the presentation of children with CHIKV in outpatient settings is mild to moderate, which is more similar to that of adults, with the high prevalence of arthralgia, headache, exanthema, and myalgia being the most prevalent findings.

## Data Availability

The original contributions presented in the study are included in the article/Supplementary Material, further inquiries can be directed to the corresponding authors.
